# Ambient air pollution and the prevalence of rhinoconjunctivitis in adolescents: a worldwide ecological analysis

**DOI:** 10.1007/s11869-018-0582-4

**Published:** 2018-06-23

**Authors:** Barbara K. Butland, H. Ross Anderson, Aaron van Donkelaar, Elaine Fuertes, Michael Brauer, Bert Brunekreef, Randall V. Martin, the ISAAC Phase Three Study Group

**Affiliations:** 1 Population Health Research Institute and MRC-PHE Centre for Environment and Health, St George’s, University of London, Cranmer Terrace, Tooting, London SW17 0RE UK; 2 MRC-PHE Centre for Environment and Health, King’s College London, London, UK; 3 Dalhousie University, Halifax, NS Canada; 4 Institute of Epidemiology 1, Helmholtz Zentrum München – German Research Centre for Environmental Health, Neuherberg, Germany; 5 School of Population and Public Health, The University of British Columbia, Vancouver, BC Canada; 6 Institute for Risk Assessment Sciences, Utrecht University, Utrecht, The Netherlands; 7 Julius Center for Health Sciences and Primary Care, University Medical Center Utrecht, Utrecht, The Netherlands; 8 Harvard-Smithsonian Centre for Astrophysics, Cambridge, MA USA

**Keywords:** Air pollution, Rhinoconjunctivitis, Childhood, NO_2_, Ozone, PM

## Abstract

Whether exposure to outdoor air pollution increases the prevalence of rhinoconjunctivitis in children is unclear. Using data from Phase Three of the International Study of Asthma and Allergies in childhood (ISAAC), we investigated associations of rhinoconjunctivitis prevalence in adolescents with model-based estimates of ozone, and satellite-based estimates of fine (diameter < 2.5 μm) particulate matter (PM_2.5_) and nitrogen dioxide (NO_2_). Information on rhinoconjunctivitis (defined as self-reported nose symptoms without a cold or flu accompanied by itchy watery eyes in the past 12 months) was available on 505,400 children aged 13–14 years, in 183 centres in 83 countries. Centre-level prevalence estimates were calculated and linked geographically with estimates of long-term average concentrations of NO_2_, ozone and PM_2.5_. Multi-level models were fitted adjusting for population density, climate, sex and gross national income. Information on parental smoking, truck traffic and cooking fuel was available for a restricted set of centres (77 in 36 countries). Between centres within countries, the estimated change in rhinoconjunctivitis prevalence per 100 children was 0.171 (95% confidence interval: − 0.013, 0.354) per 10% increase in PM_2.5_, 0.096 (− 0.003, 0.195) per 10% increase in NO_2_ and − 0.186 (− 0.390, 0.018) per 1 ppbV increase in ozone. Between countries, rhinoconjunctivitis prevalence was significantly negatively associated with both ozone and PM_2.5_. In the restricted dataset, the latter association became less negative following adjustment for parental smoking and open fires for cooking. In conclusion, there were no significant within-country associations of rhinoconjunctivitis prevalence with study pollutants. Negative between-country associations with PM_2.5_ and ozone require further investigation.

**Electronic supplementary material:**

The online version of this article (10.1007/s11869-018-0582-4) contains supplementary material, which is available to authorized users.

## Introduction

The International Study of Asthma and Allergies (ISAAC) is a programme of cross-sectional surveys of allergic disease in children conducted in centres across the world and based on standardised protocols (Ellwood et al. 2005, 2010). From comparisons within ISAAC study centres over a 7-year period, there is evidence that the prevalence and hence the health care burden associated with rhinoconjunctivitis is increasing (Asher et al. 2006). It is therefore important to try and establish what factors contribute to variations in disease prevalence at the population level. The notion of a link between allergic diseases and air pollution is well established, although findings from studies are inconsistent (Wyler et al. 2000; Hajat et al. 2001; Janssen et al. 2003; Lee et al. 2003; Gehring et al. 2010; Pénard-Morand et al. 2010; Carlsten and Melén 2012; Fuertes et al. 2013; Gehring et al. 2015; Burte et al. 2018). In previous analyses of ISAAC Phase Three, positive individual-level within-centre associations of rhinoconjunctivitis were reported with markers of exposure to combustion products, i.e. frequency of truck traffic (Brunekreef et al. 2009) and parental smoking (Mitchell et al. 2012). And in a previous meta-analysis of ISAAC Phase I data (restricted to 24 countries with more than one centre and using city-specific estimates of PM_10_ from the World Bank Global Model of ambient particles), an overall positive within-country association of PM_10_ with centre-level rhinoconjunctivitis prevalence was observed in children aged 13–14 years (Anderson et al. 2010). The aim of our ecological study was therefore to investigate whether ambient concentrations of nitrogen dioxide (NO_2_), fine particulate matter of aerodynamic diameter < 2.5 μm (PM_2.5_) and ozone might explain the wide variation in rhinoconjunctivitis symptom prevalence observed in adolescents in the ISAAC Phase Three study centres.

## Methods

Phase Three of ISAAC was mainly conducted between 2000 and 2003 and included surveys of children aged 6–7 and 13–14 years (Aït-Khaled et al. 2009). A priori we confined our current analyses to the surveys of children aged 13–14 years as for this age group information on the variables of interest and combinations thereof were available from a larger number of centres. The core self-completed questionnaire contained questions relating to allergic disease and was completed by 798,685 children in 233 centres in 97 countries. Based on these data, a child was considered to have rhinoconjunctivitis if they reported “a problem with sneezing or a runny or blocked nose when [they] did not have a cold or flu” in the past 12 months which was “accompanied by itchy-watery eyes”. An additional optional self-completed questionnaire, the environmental questionnaire, was used in a subset of ISAAC Phase Three centres and was completed by 358,982 children in 121 centres in 54 countries. The information obtained included markers of indoor and outdoor personal exposure to products of combustion (i.e. frequency of truck traffic in street of residence (“Never”, “Seldom”, “Frequently throughout the day”, and “Almost the whole day”), mother smokes (“Yes”, “No”), father smokes (“Yes”, “No”), usually use gas for cooking (“Yes”, “No”) and usually cook on open fires (“Yes”, “No”).

Information on gross national income (GNI) per capita for 2001 (Central Intelligence Agency 2003; World Bank 2009), population density for 2005 (Centre for International Earth Science Information Network 2005), vapour pressure, daily mean temperature, monthly precipitation (averaged over 1991–2000) (Mitchell 2004; Mitchell and Jones 2005), satellite-based estimates of annual mean ground-level PM_2.5_ (averaged over 2001–2006) (van Donkelaar et al. 2010) and NO_2_ (average for 2005) (Lamsal et al. 2008) and chemical transport model-based estimates of seasonal ground-level daily 1 hour maximum ozone (maximal 3 monthly running mean of daily maximum hourly ozone for 2005) were obtained as described in detail elsewhere (Anderson et al. 2012). Data on pollutants and population density were available at a spatial resolution of 0.1° latitude by 0.1° longitude whilst information on climate was available at a resolution of 0.5° latitude by 0.5° longitude. Information from these external sources was linked geographically to each ISAAC centre via a previously identified location grid of dimensions 0.1° latitude by 0.1° longitude (Anderson et al. 2012). The identification of location grids is described in detail elsewhere (Anderson et al. 2012), but in brief, the aim was to obtain grids that encapsulated the centre of population of each study area.

With respect to pollutants, estimates of ground-level PM_2.5_ were based on measures of aerosol optical depth from spectroradiometers on the satellite Terra (van Donkelaar et al. 2010) whilst estimates of ground-level NO_2_ were based on tropospheric NO_2_ columns derived from the Ozone Monitoring Instrument on the satellite Aura (Lamsal et al. 2008). In both cases, information on vertical pollutant profiles was provided by the GEOS-Chem chemical transport model and applied to the column value retrieved from the satellite instruments (Lamsal et al. 2008; van Donkelaar et al. 2010). Three-month running averages of daily 1 hour maximum ozone were derived from the TM5 chemical transport model and extrapolated to a finer spatial resolution using linear interpolation (Krol et al. 2005).

### Sample attrition

As our focus was on air pollution, which may vary substantially over relatively small areas, centres that sampled children from widely dispersed schools (centre not broadly contained within 1000 km^2^) were deliberately excluded from our analyses (Anderson et al. 2012). As a result, our principal analyses were based on 183 centres in 83 countries, all of which had complete centre-level information on rhinoconjunctivitis prevalence, sex, pollutants, climate, population density and GNI per capita.

Individual level data on sex, truck traffic, parental smoking and cooking fuel from the environmental questionnaire were available on 215,552 subjects in 82 (of the 183) centres in 38 countries. However, for five centres, complete information on these variables was provided by less than 65% of participants. Analyses involving environmental factors were therefore based on a restricted dataset of 210,665 subjects in 77 centres in 36 countries.

### Statistical methods

**Fig. 1:**
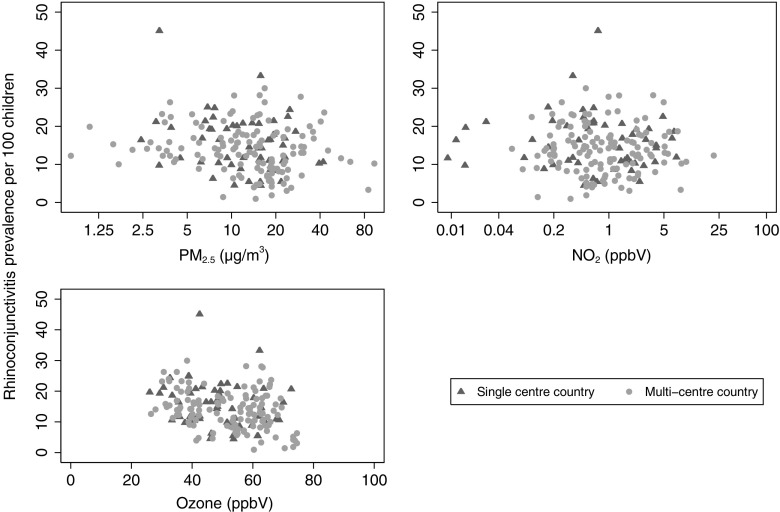
Scatterplots illustrating the association of rhinoconjunctivitis prevalence at ages 13–14 with PM_2.5_ (μg/m^3^), NO_2_ (ppbV) and ozone (ppbV) based on 183 centres in 83 countries

The calculations of all centre-level aggregates were based on individual-level data in the dataset under analysis. The associations between centre-level rhinoconjunctivitis prevalence and centre-level pollution concentrations are illustrated graphically in Fig. 1. For consistency with our previous publication (Anderson et al. 2012), we log-transformed both PM_2.5_ and NO_2_ prior to analysis. Centre-level associations between prevalence and potential confounding factors were investigated using Spearman’s correlation (see Online Resource 2 Table S1). Country-level pollution, climate and population density variables were obtained by taking an unweighted average of the corresponding centre-level variables of constituent centres.

### Two-level models (183 centres in 83 countries)

We first investigated the association between rhinoconjunctivitis prevalence and pollution at both centre (i.e. between-centre within-country) and country levels (i.e. between-country). We used two-level mixed-effects linear regression models (XTMIXED) in STATA (StataCorp 2007) with adjustment for centre-level and country-level sex, climate, population density and country-level GNI per capita. Country was modelled as a random effect and the improvement in model fit from allowing the centre-level association with pollutant to vary between countries was investigated (Begg and Parides 2003; Steele 2009). Meta-analysis regression (METAREG) in STATA was used to investigate the effect of adjusting for differences in sample size between centres (Pattenden et al. 2000).

### Two-level models (210,665 individuals in 77 centres)

Using the restricted dataset of 210,665 subjects in 77 centres, two-level (centre, individual) mixed-effects logistic regression models were fitted to the individual-level data. All models included GNI per capita (country-level): temperature, water vapour pressure, precipitation and population density at centre-level and maternal smoking, paternal smoking, gas for cooking, open fires for cooking, frequent truck traffic and sex at the individual level. Cross-level interactions between centre-level pollutants and individual-level exposures to combustion products were introduced one at a time and any improvement in model fit assessed using likelihood ratio tests. Odds ratios and 95% confidence intervals are presented with the potential effect modifier set equal to its 25th and 75th percentiles.

## Results

The following describes the results from fitting single pollutant models having adjusted for centre- and country-level sex, population density and climate and country-level GNI per capita (Table 1, model 3). For log_e_(PM_2.5_), there was little evidence of a between-centre within-country association with the prevalence of rhinoconjunctivitis, although there was evidence of a negative association between countries. The estimated difference in country-level prevalence per 10% higher country-level PM_2.5_ concentration was − 0.379 (95% CI − 0.600 to − 0.159) per 100 children. For log_e_(NO_2_), there was some suggestion of a small but not statistically significant (*p* = 0.057) positive centre-level (i.e. between-centre within-country) association with prevalence but no evidence of an association at country level (i.e. between countries). For ozone, there was no evidence of an association at centre level but a negative association at country level. The estimated difference in country-level prevalence per 1 ppbV higher country-level ozone was − 0.173 (95% CI − 0.305 to − 0.041) per 100 children.

**Table 1:** The association of rhinoconjunctivitis prevalence ages 13–14 years with PM_2.5_, NO_2_ and ozone

Model no.	Adjustment	Estimated change in rhinoconjunctivitis prevalence (95% CI) per 100 children per 10% increase in				Estimated change in rhinoconjunctivitis prevalence (95% CI) per 100 children per 1 ppbV increase in	
		PM_2.5_		NO_2_		Ozone	
		Between-centre within-country	Between-country	Between-centre within-country	Between-country	Between-centre within-country	Between-country
Using data from 183 centres in 83 countries^a^							
1	Unadjusted	0.136 (− 0.032 to 0.305)	− 0.300** (− 0.505 to − 0.096)	0.018 (− 0.067 to 0.104)	− 0.031 (− 0.133 to 0.071)	− 0.242* (− 0.441 to − 0.044)	− 0.156* (− 0.278 to − 0.034)
2	Sex, climate, GNI	0.051 (− 0.120 to 0.223)	− 0.319** (− 0.524 to − 0.114)	0.004 (− 0.079 to 0.086)	0.030 (− 0.100 to 0.160)	− 0.238* (− 0.436 to − 0.040)	− 0.171* (− 0.302 to − 0.040)
3	Sex, climate, GNI, population density	0.171 (− 0.013 to 0.354)	− 0.379*** (− 0.600 to − 0.159)	0.096 (− 0.003 to 0.195)	0.036 (− 0.107 to 0.179)	− 0.186 (− 0.390 to 0.018)	− 0.173* (− 0.305 to − 0.041)
4	Sex, climate, GNI, population density + log(NO_2_) [but log(PM_2.5_) if log(NO_2_) already in the model]	0.111 (− 0.092 to 0.315)	− 0.556*** (− 0.809 to − 0.304)	0.067 (− 0.044 to 0.178)	0.208** (0.054 to 0.362)	− 0.199 (− 0.399 to 0.000)	− 0.185** (− 0.320 to − 0.051)
5	Sex, climate, GNI, population density + the two other pollutants	0.114 (− 0.086 to 0.314)	− 0.521*** (− 0.830 to − 0.213)	0.073 (− 0.036 to 0.182)	0.204** (0.049 to 0.359)	− 0.200* (− 0.396 to − 0.005)	− 0.032 (− 0.189 to 0.125)

^a^Based on the 128 centres of the 28 countries with ≥ 2 centres and having adjusted for sex, population density and GNI per capita as in model 3; the test for a random slope in log_e_(PM_2.5_) was non-significant (*χ*^2^ = 4.31 (degrees of freedom = 2), *p* > 0.05) as was the test for a random slope in log_e_(NO_2_) (*χ*^2^ = 0.13 (degrees of freedom = 2), *p* > 0.05) and the test for a random slope in ozone (*χ*^2^ = 0.004 (degrees of freedom = 2), *p* > 0.05). All analyses are therefore based on 183 centres in 83 countries (although only countries with ≥ 2 centres provide any information on between-centre within-country associations)

*p < 0.05, **p < 0.01, ***p < 0.001

When all three pollutants were included in the same model (Table 1, model 5), the negative country-level association with log_e_(PM_2.5_) persisted and increased in magnitude whilst the negative country-level association with ozone was reduced in magnitude and no longer statistically significant. The three pollutant model also resulted in a significant positive country-level association between rhinoconjunctivitis and log_e_(NO_2_) and a significant negative centre-level association with ozone.

In modelling the relationship between prevalence and pollution using two-level mixed-effects linear regression rather than two-level mixed-effects logistic regression, we avoided the problems of over-dispersion associated with the latter but failed to adjust for differences in sample size between centres. For the full data set (i.e. 183 centres), the median sample size was 3007 (interquartile range 2341 to 3181). However, the smallest sample size was 66 and the largest 6378. We therefore re-estimated the between-centre within-country associations in Table 1, model 3, adjusting for sample size using METAREG in STATA. As a result, estimates (estimated difference in prevalence per 100 children per incremental increase in pollutant) changed almost imperceptibly from 0.171 (− 0.013 to 0.354) per 10% PM_2.5_ to 0.174 (− 0.017 to 0.364), from 0.096 (− 0.003 to 0.195) per 10% NO_2_ to 0.097 (− 0.007 to 0.200) and from − 0.186 (− 0.390 to 0.018) per 1 ppbV ozone to − 0.186 (− 0.397 to 0.024).

Information on markers of exposure to combustion products was available for a sub-set of centres (i.e. this restricted analysis was based on 77 centres in 36 countries rather than 183 centres in 83 countries). At the country-level rhinoconjunctivitis prevalence was strongly negatively correlated with paternal smoking (Spearman’s *r* = − 0.35; *p* < 0.05) and open fires for cooking (*r* = − 0.48; *p* < 0.01). Correlations with maternal smoking (*r* = 0.11), frequent truck traffic (*r* = 0.19) and gas for cooking (*r* = 0.30) were positive but non-significant.

When we additionally adjusted associations between rhinoconjunctivitis prevalence and pollution for centre- and country-level maternal smoking, paternal smoking, frequent truck traffic, gas cooking and open fires for cooking (Table 2), the estimated difference in country-level rhinoconjunctivitis prevalence per 100 children per 10% higher country-level PM_2.5_ was reduced in absolute magnitude from − 0.208 (− 0.567 to 0.151) to 0.024 (− 0.330 to 0.378).

**Table 2:** The association of rhinoconjunctivitis prevalence and pollution in children ages 13–14 years: adjusting for exposure to combustion products

Adjustment	Estimated change in rhinoconjunctivitis prevalence (95% CI) per 100 children per 10% increase in				Estimated change in rhinoconjunctivitis prevalence (95% CI) per 100 children per 1 ppbV increase in	
	PM_2.5_		NO_2_		Ozone	
	Between-centre within-country	Between-country	Between-centre within-country	Between-country	Between-centre within-country	Between-country
Using data from 183 centres in 83 countries						
Sex, climate, population density, GNI	0.171 (− 0.013 to 0.354)	− 0.379*** (− 0.600 to − 0.159**)**	0.096 (− 0.003 to 0.195)	0.036 (− 0.107 to 0.179)	− 0.186 (− 0.390 to 0.018)	− 0.173* (− 0.305 to − 0.041)
Using data from 77 centres in 36 countries						
Sex, climate, population density, GNI	0.169 (− 0.183 to 0.520)	− 0.208 (− 0.567 to 0.151)	0.108 (− 0.053 to 0.269)	0.127 (− 0.171 to 0.425)	− 0.301* (− 0.580 to − 0.022)	− 0.231* (− 0.408 to − 0.054)
Sex, climate, population density, GNI, paternal smoking, open fires for cooking	0.141 (− 0.232 to 0.514)	− 0.085 (− 0.456 to 0.287)	0.095 (− 0.074 to 0.264)	0.218 (− 0.085 to 0.521)	− 0.278 (− 0.566 to 0.010)	− 0.210* (− 0.390 to − 0.029)
Sex, climate, population density, GNI, paternal smoking, open fires for cooking, maternal smoking	0.175 (− 0.223 to 0.573)	− 0.023 (− 0.404 to 0.358)	0.120 (− 0.063 to 0.304)	0.208 (− 0.090 to 0.506)	− 0.283 (− 0.576 to 0.010)	− 0.194* (− 0.386 to − 0.003)
Sex, climate, population density, GNI, paternal smoking, open fires for cooking, maternal smoking, frequent truck traffic, gas for cooking	0.139 (− 0.273 to 0.550)	0.024 (− 0.330 to 0.378)	0.117 (− 0.070 to 0.304)	0.194 (− 0.083 to 0.471)	− 0.245 (− 0.561 to 0.072)	− 0.154 (− 0.346 to 0.039)

*p < 0.05, **p < 0.01, ***p < 0.001

Finally, Table 3 investigates whether individual-level associations between rhinoconjunctivitis and exposure to combustion products are modified by centre-level pollution. For both log_e_(PM_2.5_) and ozone, odds ratios for exposure to frequent truck traffic and paternal smoking were marginally but significantly higher in centres with high rather than low background concentrations, although for the full truck-traffic variable (see Online Resource 2 Table S2), the pattern of any effect modification was not consistent across categories. A significant cross-level interaction was also observed between gas cooking and log_e_(NO_2_), although in the absence of any association at the individual level (Wong et al. 2013). This interaction is difficult to interpret and may be spurious.

**Table 3:** Investigating the effect of centre-level pollution variables on the individual-level associations between rhinoconjunctivitis and exposure to combustion products

Exposure (yes vs no)	Potential effect modifier								
	log_e_(PM_2.5_) set equal to its		Test for effect modification by log_e_(PM_2.5_)	log_e_(NO_2_) set equal to its		Test for effect modification by log_e_(NO_2_)	Ozone set equal to its:		Test for effect modification by ozone
	25th percentile	75th percentile		25th percentile	75th percentile		25th percentile	75th percentile	
	OR (95% CI)	OR (95% CI)		OR (95% CI)	OR (95% CI)		OR (95% CI)	OR (95% CI)	
Using data from 210,665 individuals in 77 centres									
Mother smokes	1.15 (1.11 to 1.20)	1.20 (1.14 to 1.25)	*p* = 0.093	1.18 (1.13 to 1.25)	1.16 (1.11 to 1.21)	*p* = 0.410	1.15 (1.10 to 1.20)	1.20 (1.14 to 1.26)	*p* = 0.177
Father smokes	1.09 (1.05 to 1.12)	1.14 (1.10 to 1.18)	*p* = 0.002	1.13 (1.09 to 1.17)	1.10 (1.06 to 1.14)	*p* = 0.224	1.08 (1.05 to 1.12)	1.14 (1.10 to 1.19)	*p* = 0.025
Frequent truck traffic	1.24 (1.20 to 1.28)	1.30 (1.26 to 1.34)	*p* = 0.003	1.25 (1.21 to 1.29)	1.28 (1.24 to 1.32)	*p* = 0.255	1.23 (1.19 to 1.27)	1.31 (1.26 to 1.36)	*p* = 0.005
Gas for cooking	0.98 (0.93 to 1.02)	1.01 (0.97 to 1.06)	*p* = 0.140	0.95 (0.91 to 1.00)	1.02 (0.98 to 1.07)	*p* = 0.018	0.98 (0.93 to 1.03)	1.01 (0.96 to 1.06)	*p* = 0.374
Open fires for cooking	1.22 (1.12 to 1.32)	1.27 (1.17 to 1.37)	*p* = 0.184	1.27 (1.17 to 1.38)	1.21 (1.09 to 1.34)	*p* = 0.343	1.25 (1.13 to 1.39)	1.25 (1.15 to 1.37)	*p* = 0.970

All models include GNI per capita at country level; temperature, water vapour pressure, precipitation and population density at centre level; and maternal smoking, paternal smoking, gas for cooking, open fires for cooking, frequent truck traffic and sex at the individual level

## Discussion

### Main findings

In our ecological analysis of the association between the prevalence of rhinoconjunctivitis and yearly pollution concentration adjusted for centre- and country-level sex, climate and population density and country-level GNI, we found evidence of differences in centre-level and country-level associations. At centre level, associations with both log_e_(PM_2.5_) and log_e_(NO_2_) though positive were small and not statistically significant, whilst at country level, there were significant negative associations of rhinoconjunctivitis prevalence with both log_e_(PM_2.5_) and ozone.

### Centre-level associations

Evidence of a link between air pollution, particularly diesel exhaust particles, and allergic sensitisation comes from experimental (Diaz-Sanchez et al. 2000; Carlsten and Melén 2012) and epidemiological studies (Wyler et al. 2000; Janssen et al. 2003; Pénard-Morand et al. 2010). It is thought that air pollutant exposure may induce oxidative stress leading to inflammation and facilitating the enhanced presentation of allergens to mast cells, which in turn results in an increase in histamine release and the severity of allergy-related symptoms (Diaz-Sanchez et al. 2000; Saxon and Diaz-Sanchez 2005; Carlsten and Melén 2012). This sort of mechanism suggests that short-term (e.g. day to day) as well as long-term (e.g. annual average) pollutant exposures may have a role to play in the pathogenesis of rhinoconjunctivitis.

Outside of the ISAAC programme, however, there are relatively few epidemiological studies of air pollution or air pollution markers considering hay fever or rhinoconjunctivitis as outcomes and the findings are inconsistent (Wyler et al. 2000; Hajat et al. 2001; Janssen et al. 2003; Lee et al. 2003; Gehring et al. 2010; Pénard-Morand et al. 2010; Fuertes et al. 2013; Gehring et al. 2015; Burte et al. 2018). Nevertheless, in Taiwan, a large study of 312,873 middle-school children within 55 communities reported a weak negative community-level association of allergic rhinitis prevalence with annual average ozone, which the authors suggested might be due to scavenging by traffic exhaust emissions and a positive community-level association with annual average NO_x_ (oxides of nitrogen) (Lee et al. 2003). Similarly, a study of over 2000 children aged 7–12 years in 24 Dutch schools situated close to motorways reported a positive association of hay fever ever with school-level annual average PM_2.5_ and positive associations of current conjunctivitis (i.e. in the past 12 months) with both school-level annual average NO_2_ and PM_2.5_ (Janssen et al. 2003). Though consistent in direction with our own centre-level findings, in our study, there were small positive but not statistically significant associations with log_e_(NO_2_) and log_e_(PM_2.5_) and a small negative and not significant association with ozone. Further, between-community associations (e.g. centre-level, school-level) may be very different to those at the individual or within-community level. In a large time-series study of London children, a strong positive (rather than negative) association was observed between ozone (averaged over 0–3 days prior) and general practice consultations for rhinoconjunctivitis, although the focus here was on the exacerbation of symptoms rather than prevalence (Hajat et al. 2001).

### Country-level associations

From our two and three pollutant models in Table 1, it would appear that at country level, the negative association with ozone may be explained by the negative association with log_e_(PM_2.5_). This is not surprising given the strong country-level correlation (Spearman *r* = 0.56; *p* = 0.0004) between the two pollutants. However, the dominance of one association over the other could possibly result from the different uncertainties associated with each pollutant. Negative country-level associations with these two pollutants have previously been observed in ISAAC Phase Three with severe asthma prevalence (Anderson et al. 2012). Such associations may therefore be driven by some factor or factors common to allergic disease rather than specific to rhinoconjunctivitis. They are nevertheless at odds with positive individual-level associations observed in ISAAC Phase Three: between both rhinoconjunctivitis and severe asthma and exposure to paternal smoking, maternal smoking (Mitchell et al. 2012) and frequent truck traffic (Brunekreef et al. 2009) and between severe asthma and the use of open fires for cooking (Wong et al. 2013). When we adjusted our analyses for these exposures (i.e. their averages at centre and country level), we found some evidence that the negative country-level association of log_e_(PM_2.5_) with rhinoconjunctivitis (Table 2) was explained by country-level parental smoking (particularly paternal smoking) and country-level open fires for cooking.

Mitchell et al. (2012) have already shown that, whereas at the individual level, there is evidence in ISAAC Phase Three that paternal smoking is positively associated with symptoms of asthma and rhinoconjunctivitis in adolescents; at an ecological level in ISAAC Phase One (i.e. across centres, adjusted for country-level GNP), the relationship is negative (Mitchell et al. 2001). In our current study, we observed strong negative correlations of rhinoconjunctivitis prevalence with both paternal smoking and open fires for cooking at country level.

As we observed significant positive associations at the individual level and negative relationships at the country level, this suggests an important role for other factors, besides pollution, on country-level prevalence. Centre-level disease prevalence depends not only on the risk associated with a given exposure at the individual level or the proportion of the centre population exposed but also on the baseline risk, which is the risk of disease due to other exposures and genetic predisposition.

Whilst there may be further scope in linking ISAAC data with other global databases in order to try and explain differences in country-level disease prevalence, these differences may themselves suggest novel risk factors or risk factor interactions to be investigated in future epidemiological studies.

### Study limitations

Ambient air pollution is a universal exposure; i.e. all in the population are exposed to some extent. However, the level of that exposure will vary depending on factors such as time spent indoors, type of building, proximity to roads and distance of home from school. Our pollution data, whether model based or satellite based, estimate outdoor background ground-level exposure at centre level and may therefore tell us little about individual-level exposure, in particular to components which occur in the indoor environment. Nevertheless, we found some evidence that in centres with higher levels of PM_2.5_ and ozone, previously reported positive individual-level associations between rhinoconjunctivitis and both frequent truck traffic (Brunekreef et al. 2009), and paternal smoking (Mitchell et al. 2012), were slightly more marked (Table 3).

In interpreting between-country associations, we need to be careful as our measures of country-level prevalence are not based on a representative sample of 13–14-year-old children from that country. However, our country-level associations are of interest only as signposts to any real causes by which they may be “confounded”. Our focus is therefore on associations between centres within countries as given our method of analysis, these are adjusted for country-level differences both measured and unmeasured. These centre-level associations are effectively based on data from 128 centres in 28 countries (i.e. countries with at least two centres), 16 of which provided data from at least 3 centres (i.e. New Zealand (3 centres), Spain (9), Italy (9), Portugal (4), Serbia and Montenegro (5), Iran (4), Chile (4), Brazil (19), Argentina (4), Mexico (9), China (5), India (16), Thailand (4), Kyrgyzstan (3), Lithuania (3), Syria (3)). Our findings may therefore be disproportionately influenced by associations within Brazil and India but also to a lesser extent by associations in Spain, Italy and Mexico.

## Conclusion

In our global ecological analysis, we found no evidence of centre-level associations between rhinoconjuntivitis and pollutants. The observed negative country-level associations likely reflect complex relationships involving genetic and multiple social, demographic and environmental factors rather than exposure to air pollution.

## Electronic supplementary material

ESM 1 (DOCX 29 kb)

ESM 2 (DOCX 25 kb)
