# Supplementary material for: Ambient air pollution and the prevalence of rhinoconjunctivitis in adolescents: a worldwide ecological analysis
Source: Air Qual Atmos Health. 2018 Jun 23;11(7):755–64. doi: 10.1007/s11869-018-0582-4 (PMC6097066; doi:10.1007/s11869-018-0582-4)
Supplement: Supplementary file 1 — (DOCX 29 kb) [file 11869_2018_582_MOESM1_ESM.docx]

**Online Resource 1** Listing of the ISAAC Phase III Study Group

Supplementary material for: Butland BK [b.butland@sgul.ac.uk](mailto:b.butland@sgul.ac.uk)^1^, Anderson HR^1,2^, van Donkelaar A^3^, Fuertes E^4^, Brauer M^5^, Brunekreef B^6,7^, Martin RV^3,8^, and the ISAAC Phase Three Study Group^9^. Ambient air pollution and the prevalence of rhinoconjunctivitis in adolescents: A worldwide ecological analysis. (Submitted to: Air Quality, Atmosphere & Health)

^1^Population Health Research Institute and MRC-PHE Centre for Environment and Health, St George’s, University of London, UK; ^2^MRC-PHE Centre for Environment and Health, King’s College London, UK; ^3^Dalhousie University, Halifax, NS, Canada; ^4^Institute of Epidemiology 1, Helmholtz Zentrum München – German Research Centre for Environmental Health Neuherberg, Germany; ^5^School of Population and Public Health, The University of British Columbia, Vancouver, BC, Canada; ^6^Institute for Risk Assessment Sciences, Utrecht University, Utrecht, The Netherlands; ^7^Julius Center for Health Sciences and Primary Care, University Medical Center Utrecht, Utrecht, The Netherlands; ^8^Harvard-Smithsonian Centre for Astrophysics, Cambridge, Massachusetts, USA; ^9^ISAAC Phase Three Study Group listed below.

**ISAAC Phase Three Study Group**

***ISAAC Steering Committee:***

N Aït-Khaled* (International Union Against Tuberculosis and Lung Diseases, Paris, France); HR Anderson (Division of Community Health Sciences, St Georges, University of London, London, UK); MI Asher (Department of Paediatrics: Child and Youth Health, Faculty of Medical and Health Sciences, The University of Auckland, New Zealand); R Beasley* (Medical Research Institute of New Zealand, Wellington, New Zealand); B Björkstén* (Institute of Environmental Medicine, Karolinska Institutet, Stockholm, Sweden); B Brunekreef (Institute of Risk Assessment Science, Universiteit Utrecht, Netherlands); J Crane (Wellington Asthma Research Group, Wellington School of Medicine, New Zealand); P Ellwood (Department of Paediatrics: Child and Youth Health, Faculty of Medical and Health Sciences, The University of Auckland, New Zealand); C Flohr (Department of Paediatric Allergy & Dermatology, St John’s Institute of Dermatology, London, UK); S Foliaki* (Centre for Public Health Research, Massey University, Wellington, New Zealand); F Forastiere (Department of Epidemiology, Rome E Health Authority, Rome, Italy); L García-Marcos (Respiratory Medicine and Allergy Units, 'Virgen de la Arrixaca' University Children's Hospital, University of Murcia, Spain); U Keil* (Institut für Epidemiologie und Sozialmedizin, Universität Münster, Germany); CKW Lai* (Department of Medicine and Therapeutics, The Chinese University of Hong Kong SAR, China); J Mallol* (Department of Paediatric Respiratory Medicine, University of Santiago de Chile, Chile); EA Mitchell (Department of Paediatrics: Child and Youth Health, Faculty of Medical and Health Sciences, The University of Auckland, New Zealand); S Montefort* (Department of Medicine, University of Malta, Malta), J Odhiambo*† (Centre Respiratory Diseases Research Unit, Kenya Medical Research Institute, Nairobi, Kenya); N Pearce (London School of Hygiene and Tropical Medicine, London, UK and Centre for Public Health Research, Massey University, Wellington, New Zealand); CF Robertson (Murdoch Children's Research Institute, Melbourne, Australia); AW Stewart (Population Health, Faculty of Medical and Health Sciences, The University of Auckland, New Zealand); D Strachan (Division of Community Health Sciences, St Georges, University of London, London, UK); E von Mutius (Dr von Haunerschen Kinderklinik de Universität München, Germany); SK Weiland† (Department of Epidemiology, University of Ulm, Germany); G Weinmayr (Institute of Epidemiology, University of Ulm, Germany); H Williams (Centre for Evidence Based Dermatology, Queen’s Medical Centre, University Hospital, Nottingham, UK); G Wong (Department of Paediatrics, Prince of Wales Hospital, Hong Kong SAR, China).

* Regional Coordinator; † Deceased.

***ISAAC International Data Centre:***

MI Asher, TO Clayton†, E Ellwood, P Ellwood, EA Mitchell, Department of Paediatrics: Child and Youth Health, and AW Stewart, School of Population Health, Faculty of Medical and Health Sciences, The University of Auckland, New Zealand.

† Deceased

***ISAAC Phase Three Principal Investigators:***

**Albania:** A Priftanji***** - Mother Theresa University Hospital of Tirana (Tiranë); **Algeria:** B Benhabylès - CHU Mustapha (Wilaya of Algiers); **Argentina:** CE Baena-Cagnani***†** - Catholic University of Córdoba (Córdoba), CD Crisci - National University of Rosario (Rosario City), M Gómez - Hospital San Bernardo (Salta), GE Zabert - Medicina y Cirugía-Escuela de Medicina-UNComahue (Neuquén); **Australia:** CF Robertson***** - Murdoch Children's Research Institute (Melbourne); **Austria:** G Haidinger***** - Medical University Vienna (Urfahr-Umgebung); **Barbados:** ME Howitt***** - Carlton Clinic (Barbados); Belgium: J Weyler - University of Antwerp (Antwerp); **Bolivia:** R Pinto-Vargas***** - Caja Petrolera de Salud (Santa Cruz); **Brasil:** Cd Bernhardt - Universidade do Vale do Itajaí (Itajaí), WG Borges - Hospita da Crianca de Brasilia Jose Alencar (Brasília), PA Camargos - Federal University of Minas Gerais (Belo Horizonte), Md Cardoso - Rua Paraiba, 1020 (Manaus Amazonas), AJ da Cunha - Federal University of Rio de Janeiro (Nova Iguaçu), M de Britto - Instiuto de Medicina Integral (Recife), L de Freitas Souza - Universidade Federal da Bahia (Feira de Santana, Salvador, Vitória da Conquista), GB Fischer - Universidad Federal (Porto Alegre), JM Motta - (Aracaju), FJ Passos Soares - Alagoas Federal University (Maceió), AC Pastorino - (São Paulo West), AC Porto Neto - Passo Fundo University (Passo Fundo), N Rosário - University of Parana (Curitiba), A Silva - UFPE (Caruaru), D Solé***** - Universidade Federal de São Paulo (São Paulo, Santa Maria), N Wandalsen - Faculdadade de Medicina do ABC (Santo Andre); **Bulgaria:** T Popov***** - SUH ‘Alexandrovska’ Medical University (Sofia); **Cameroon:** C Kuaban - University of Yaounde (Yaounde); **Canada:** A Ferguson - University of British Columbia (Vancouver); **Channel Islands:** R Goulding - Policy Principal (Jersey), P Standring - Princess Elizabeth Hospital (Guernsey); **Chile:** P Aguilar - Hospital CRS El Pino (South Santiago), L Amarales - Regional Hospital "Lautaro Navarro" (Punta Arenas), LA Benavides - (Calama), MA Calvo - Universidad Austral de Chile (Valdivia); **China:** Y-Z Chen***** - Training Hospital for Peking University (Beijing, Tong Zhou), O Kunii - University of Tokyo (Tibet), Q Li Pan - Xinjiang Children's Hospital (Wulumuqi), N-S Zhong - Guangzhou Institute of Respiratory Disease (Guangzhou); **Colombia:** AM Cepeda - Universidad Metropolitana (Barranquilla), GA Ordoñez - Universidad Libre de Cali (Cali); **Congo:** J M'Boussa - Centre Hospitalier Universitaire (Brazzaville); **Cook Islands:** R Daniel***** - Ministry of Health, Cook Islands (Rarotonga); **Croatia:** K Lah Tomulic - Children Hospital Kantrida (Rijeka); **Cuba:** P Varona Peréz***** - Instituto Naconal de Higiene Epidemiología y Microbiología (La Habana); **Ecuador:** S Barba***** - AXXIS-Medical Centre SEAICA (Quito), C Bustos - Hospital Alcivar (Guayaquil); **Egypt:** ML Naguib - Cairo University Children's Hospital (Cairo); **El Salvador:** M Figueroa Colorado***** - Universidad Dr Jose Matias Delgado (San Salvador); **Estonia:** M-A Riikjärv***** - Tallinn Children's Hospital (Tallinn); **Ethiopia:** K Melaku - Addis Ababa University (Addis Ababa); **Former Yugoslav Republic Of Macedonia (FYROM):** E Vlaski***** - University Children's Hospital (Skopje); **Gabon:** IE Hypolite***** - (Port-Gentil); **Georgia:** M Gotua***** - Center of Allergy & Immunology (Kutaisi); **Germany:** U Keil***** - Westfälische Wilhelms Universität (Münster); **Honduras:** A Bueso-Engelhardt***** - Centro de Neumología y Alergia (San Pedro Sula); **Hong Kong:** G Wong - Prince of Wales Hospital (Hong Kong 13-14), **Hungary:** Z Novák - University of Szeged (Szeged); **India:** S Bhave - KEM Hospital Research Centre (Rasta Peth), J Chhatwal - Christian Medical College and Hospital (Ludhiana), NM Hanumante - Ruby Hall Clinic (Pune), KC Jain - Pioneer Medical Centre (Jodhpur), VA Khatav - Dr Khatav's Mother and Child Hospital (Borivali), L Kumar - Professor & Former Head (Chandigarh), SN Mantri - C/- Dr J.R. Shah (Mumbai (29)), AV Pherwani - P.D. Hinduja National Hospital (Mumbai (18)), M Sabir - Respiratory Division, Department of Medicine (Bikaner), S Salvi - Chest Research Foundation (Nagpur, Pimpri), G Setty - (Chennai), SK Sharma - All India Institute of Medical Sciences (New Delhi (7)), V Singh - Asthma Bhawan, Jaipur (Jaipur), T Sukumaran - Pushpagiri Medical College (Kottayam), PS Suresh Babu - Bapuji Child Health Institute and Research Centre (Davangere); **Indonesia:** CB Kartasasmita - Padjajaran University (Bandung), W Suprihati - Diponegoro University (Semarang); **Iran:** M-R Masjedi***** - National Research Institute of Tuberculosis and Lung Diseases (Birjand, Rasht, Tehran, Zanjan); **Isle Of Man:** A Steriu - Public Health Specialist, Information and Research (Isle of Man); **Italy:** L Armenio - Clinica Pediatrica III dell'Università di Bari (Bari), L Bisanti - ASL Città di Milano (Milano), E Bonci - Università degli Studi di Roma "La Sapienza" (Cosenza), E Chellini - Istituto per lo Studio e la Prevenzione Oncologica (Firenze), G Ciccone - S. Giovanni Battista Hospital (Torino), V Dell'Orco - Local Health Unit, ASL (Colleferro-Tivoli), F Forastiere***** - Rome E Health Authority (Roma), S La Grutta - Institute of Biomedicine and Molecular Immunology (Palermo), MG Petronio - Local Health Authority (Empoli); **Japan:** H Odajima - National Hospital Organization Fukuoka Hospital (Fukuoka), M Sohei - Dokkyo University School of Medicine (Tochigi); **Jordan:** F Abu-Ekteish - Jordon University of Science and Technology (Amman); **Kenya:** FO Esamai - Moi University College of Health Sciences (Eldoret), L Ng’ang’a***** - Centres for Disease Control & Prevention (Nairobi); **Kuwait:** JA al-Momen - Al-Amiri Hospital (Kuwait); **Kyrgyzstan:** C Imanalieva***** - Kyrgyz Scientific Research Institute of Obstetrics and Pediatrics (Balykchi, Bishkek), S Sulaimanov - Osh State University (Jalalabat); **Latvia:** V Svabe - Riga Strading University (Riga); **Lithuania:** J Bojarskas - Kaunas Medical University (Panevezys, Siauliai), J Kudzyte***** - Kaunas Medical University (Kaunas); **Malaysia:** BS Quah - Melaka-Manipal Medical College, (Kota Bharu), KH Teh - Hospital Alor Setar (Alor Setar); **Malta:** S Montefort***** - University of Malta (Malta); **Mexico:** M Baeza-Bacab***** - University Autónoma de Yucatán (Mérida), M Barragán-Meijueiro - CoMAAIPE (Ciudad de México (3)), BE Del-Río-Navarro - Hospital Infantil de México (Ciudad de México (1)), R García-Almaráz - Hospital Infantil de Tamaulipas (Ciudad Victoria), FJ Linares-Zapién - Centro De Enfermedades Alergicas Y Asma de Toluca (Toluca), JV Merida-Palacio - Centro de Investigacion de Enfermedades Alergicas y Respiratorias (Mexicali Valley), N Ramírez-Chanona - CoMAIPE (Ciudad de México (4)), S Romero-Tapia - Hospital de Alta Especialidad del Niño (Villahermosa), I Romieu - International Agency for Research on Cancer (Cuernavaca); **Morocco:** Z Bouayad***** - Hôpital 20 Août (Casablanca, Marrakech); **New Zealand:** R MacKay - Canterbury Health Laboratories (Nelson), P Pattemore - University of Otago, Christchurch (Christchurch), N Pearce - London School of Hygiene and Tropical Medicine (Wellington); **Nicaragua:** JF Sánchez***** - Hospital Infantil Manuel de Jesús Rivera (Managua); **Nigeria:** BO Onadeko - (Ibadan); **Niue:** M Magatogia - Niue Foou Hospital (Niue Island); **Pakistan:** N Mahmood***** - The Aga Khan University (Karachi),  MO Yusuf - The Allergy & Asthma Institute (Islamabad); **Palestine:** N El Sharif***** - Al Quds University (Ramallah), S Mortaja - Al Quds University-Gaza Branch (North Gaza); **Paraguay:** JA Guggiari-Chase***** - Centro Médico Bautista (Asunción); **Peru:** P Chiarella***** - Universidad Peruana de Ciencias Aplicadas, UPC (Lima); **Philippines:** F Cua-Lim***** - University of Santo Tomas (Metro Manila); **Poland:** A Brêborowicz - University of Medical Sciences (Poznan), G Lis***** - Jagiellonian University (Kraków); **Portugal:** R Câmara - Centro Hospitilar do Funchal (Funchal), ML Chiera - Hosp. Ped. Coimbra (Coimbra), JM Lopes dos Santos - Hospital Pedro Hispano (Porto), C Nunes - Center of Allergy and Immunology of Algarve (Portimao); **République De Guinée:** OY Sow - Centre Hospitalier Universitaire Ignace Deen (Conakry); **Republique Democratique Du Congo:** J-M Kayembe - Université de Kinshasa (Kinshasa); **Romania**: D Deleanu***** - University of Medicine & Pharmacy IULIU Hatieganu (Cluj); **Russia:** EG Kondiourina - Novosibirsk State Medical University (Novosibirsk); **Samoa:** P Fuimaono V Pisi - (Apia); **Serbia And Montenegro:** O Adzovic - Children's Hospital (Podgorica), M Hadnadjev - Primary Health Care (Novi Sad), E Panic - Regional Health Care Centre (Sombor), S Zivanovic - Pediatric Clinic (Nis), Z Zivkovic***** - Children’s Hospital for Lung Diseases and Tuberculosis (Belgrade); **Singapore:** DYT Goh - National University of Singapore (Singapore); **South Korea:** H-B Lee***** - Hanyang University College of Medicine (Seoul); **Spain:** A Arnedo-Pena - Center Public Health Castellon (Castellón), RM Busquets - Metge Adjunt, Unitat de Pneumologia Pediatica, (Barcelona), G García-Hernández - Hospital Universitario 12 de Octubre (Madrid), L García-Marcos***** - 'Virgen de la Arrixaca' University Children's Hospital (Cartagena), C González Díaz - Universidad del País Vasco UPV /EHU (Bilbao), F Guillén-Grima - UPNA (Pamplona), A López-Silvarrey Varela - Fundacion Maria Jose Jove (A Coruña), M Morales Suárez-Varela - Valencia University-CIBERESP (Valencia), EG Pérez-Yarza - Universidad del Pais Vasco UPV/EHU (San Sebastián); **Sweden:** H Vogt - Linköping University (Linköping); **Syria:** Y Mohammad - National Center for Research and Training in Chronic Respiratory Diseases - Tishreen University (Lattakia), S Mohammad***** - Head of Paediatrics Department (Tartous), K Tabbah - Aleppo University Hospital (Aleppo); **Taiwan:** J-L Huang***** - Chang Gung University (Taipei); **Thailand:** R Nettagul - 230/7 Thanalai Road (Chiangrai), T Prasarnphanich - Prapokklao Hospital (Chantaburi), J Teeratakulpisarn - Khon Kaen University (Khon Kaen), M Trakultivakorn - Chiang Mai University (Chiang Mai); **Togo:** O Tidjani - CHU Tokoin (Lome); **Tokelau:** T Iosefa - Ministry of Health (Tokelau); **Tonga:** S Foliaki - Massey University (Nuku alofa); **Trinidad And Tobago:** MA Monteil - University of the West Indies (Tobago); **Tunisia:** M Jerray - Hopital Universitaire F. Hached (Sousse), F Khaldi - Hôpital d'Enfants Bab Saadoun (Grand Tunis); **Ukraine:** V Ognev S Mohammad***** - Kharkov National Medical University (Kharkiv); **United Kingdom:** MH Shamssain - University of Sunderland (Sunderland); **Uruguay:** D Holgado***** - Hospital Pereira Rossell (Montevideo); **Usa:** GJ Redding - Seattle Children's Hospital (Seattle); **Venezuela:** O Aldrey***** - Jefe del Instituto (Caracas); **Vietnam:** B Vaên Cam - Pediatric Hospital 1# (Ho Chi Minh City)

***** National Coordinator

**†** Deceased

***ISAAC National Coordinators not identified above:***

**Canada**: M Sears - McMaster University; **Channel Islands**: HR Anderson - St George's, University of London; **Chile**: V Aguirre - Hospital CRS El Pino; **Colombia**: J Mallol - Hospital CRS El Pino (interim); **Croatia**: V Ahel - Children's Hospital Kantrida; **Hong Kong**: CKW Lai -The Chinese University of Hong Kong; **Hungary**: G Zsigmond; **India**: J Shah -Jaslok Hospital & Research Centre; **Indonesia**: K Baratawidjaja - University of Indonesia; **Malaysia**: J de Bruyne - University of Malaya; **Netherlands**: R Otten - University of Nijmegen; **New Zealand**: MI Asher – University of Auckland; **Portugal**: J Rosado Pinto - Hospital da Luz; **Republique Democratique du Congo**: E Bahati – PMLT; **Russia**: RM Khaitov - National Research Center; **Singapore**: B-W Lee - National University Hospital; **Sweden**: L Nilsson - University Hospital, Linköping; **Thailand**: P Vichyanond - Mahidol University; **United Kingdom**: R Anderson- St George's, University of London.
