# Supplementary material for: Ambient air pollution and the prevalence of rhinoconjunctivitis in adolescents: a worldwide ecological analysis
Source: Air Qual Atmos Health. 2018 Jun 23;11(7):755–64. doi: 10.1007/s11869-018-0582-4 (PMC6097066; doi:10.1007/s11869-018-0582-4)
Supplement: Supplementary file 2 — (DOCX 25 kb) [file 11869_2018_582_MOESM2_ESM.docx]

**Online Resource 2** Additional analyses

Supplementary material for: Butland BK [b.butland@sgul.ac.uk](mailto:b.butland@sgul.ac.uk)^1^, Anderson HR^1,2^, van Donkelaar A^3^, Fuertes E^4^, Brauer M^5^, Brunekreef B^6,7^, Martin RV^3,8^, and the ISAAC Phase Three Study Group^9^. Ambient air pollution and the prevalence of rhinoconjunctivitis in adolescents: A worldwide ecological analysis. (Submitted to: Air Quality, Atmosphere & Health)

^1^Population Health Research Institute and MRC-PHE Centre for Environment and Health, St George’s, University of London, UK; ^2^MRC-PHE Centre for Environment and Health, King’s College London, UK; ^3^Dalhousie University, Halifax, NS, Canada; ^4^Institute of Epidemiology 1, Helmholtz Zentrum München – German Research Centre for Environmental Health Neuherberg, Germany; ^5^School of Population and Public Health, The University of British Columbia, Vancouver, BC, Canada; ^6^Institute for Risk Assessment Sciences, Utrecht University, Utrecht, The Netherlands; ^7^Julius Center for Health Sciences and Primary Care, University Medical Center Utrecht, Utrecht, The Netherlands; ^8^Harvard-Smithsonian Centre for Astrophysics, Cambridge, Massachusetts, USA; ^9^ISAAC Phase Three Study Group listed in Online Resource 1.

Contents:

Table S1: Investigating associations at centre-level between rhinoconjunctivitis prevalence and potential confounding factors.

Table S2: Investigating the effect of centre-level pollution variables on the individual-level associations between rhinoconjunctivitis and exposure to truck-traffic.

**Table S1** Centre-level Spearman correlation coefficients between rhinoconjunctivitis prevalence and potential confounding factors (N=183 centres).

| Centre-level confounding factor | Spearman correlation with centre-level rhinoconjunctivitis prevalence |
| --- | --- |
| Percentage of boys | -0.055 |
| Mean daily temperature (◦c) | 0.165* |
| Mean water vapour pressure (hPa) | 0.189* |
| Mean precipitation (mm/month) | 0.133 |
| Population density  (thousands per 0.1^◦^ x 0.1^◦^ grid square) | 0.012 |
| GNI per capita (country-level) | 0.218** |

*p<0.05; **p<0.01

**Table S2** Investigating the effect of centre-level pollution variables on the individual-level associations between rhinoconjunctivitis and exposure to truck-traffic^¶^

| Exposure to truck-traffic | Potential effect modifier | | | | | | | | |
| --- | --- | --- | --- | --- | --- | --- | --- | --- | --- |
|  | log_e_(PM_2.5_) set equal to its: | | Test for effect modification by log_e_(PM_2.5_) | log_e_(NO_2_) set equal to its: | | Test for effect modification by  log_e_(NO_2_) | Ozone set equal to its: | | Test for  effect modification  by  ozone |
|  | 25^th^ percentile  OR (95% CI) | 75^th^ percentile  OR (95% CI) |  | 25^th^ percentile  OR (95% CI) | 75^th^ percentile  OR (95% CI) |  | 25^th^ percentile  OR (95% CI) | 75^th^ percentile  OR (95% CI) |  |
| “Never” | 1.00  (reference) | 1.00 (reference) | p=0.010 | 1.00 (reference) | 1.00 (reference) | p=0.503 | 1.00 (reference) | 1.00 (reference) | p=0.012 |
| “Seldom” | 1.09 (1.04 to 1.14) | 1.06 (1.01 to 1.11) |  | 1.08 (1.02 to 1.13) | 1.08 (1.03 to 1.13) |  | 1.09  (1.03 to 1.15) | 1.06  (1.00,1.12) |  |
| “Frequently throughout the day” | 1.26 (1.20 to 1.32) | 1.30 (1.24 to 1.37) |  | 1.26 (1.19,1.33) | 1.30 (1.23 to 1.37) |  | 1.24  (1.17 to 1.31) | 1.32  (1.25 to 1.40) |  |
| “Almost the whole day” | 1.42 (1.35 to 1.50) | 1.44 (1.36 to 1.52) |  | 1.41 (1.33 to 1.49) | 1.45 (1.37 to 1.54) |  | 1.42  (1.34 to 1.51) | 1.44  (1.35 to 1.53) |  |
| ^¶^All models include GNI per capita at country-level; temperature, water vapour pressure, precipitation and population density at centre-level; maternal smoking, paternal smoking, gas for cooking, open fires for cooking and sex at the individual-level. | | | | | | | | | |
